# The Acute and Early Effects of Whole-Brain Irradiation on Glial Activation, Brain Metabolism, and Behavior: a Positron Emission Tomography Study

**DOI:** 10.1007/s11307-020-01483-y

**Published:** 2020-02-12

**Authors:** Andrea Parente, Erik F. J. de Vries, Aren van Waarde, Magdalini Ioannou, Peter van Luijk, Johannes A. Langendijk, Rudi A. J. O. Dierckx, Janine Doorduin

**Affiliations:** 1 Department of Nuclear Medicine and Molecular Imaging, University Medical Center Groningen, University of Groningen, Hanzeplein 1, 9713 GZ Groningen, The Netherlands; 2 Department of Cell Biology, University Medical Center Groningen, University of Groningen, Groningen, The Netherlands; 3 Department of Radiation Oncology, University Medical Center Groningen, University of Groningen, Groningen, The Netherlands

**Keywords:** Brain irradiation, Neuroinflammation, Microglia activation, Brain metabolism, Brain imaging, Behavior, PET imaging

## Abstract

**Purpose:**

Radiotherapy is a frequently applied treatment modality for brain tumors. Concomitant irradiation of normal brain tissue can induce various physiological responses. The aim of this study was to investigate whether acute and early-delayed effects of brain irradiation on glial activation and brain metabolism can be detected with positron emission tomography (PET) and whether these effects are correlated with behavioral changes.

**Procedures:**

Rats underwent 0-, 10-, or 25-Gy whole-brain irradiation. At 3 and 31 days post irradiation, 1-(2-chlorophenyl)-N-[^11^C]methyl-(1-methylpropyl)-3-isoquinoline carboxamide ([^11^C]PK11195) and 2-deoxy-2-[^18^F]fluoro-d-glucose ([^18^F]FDG) PET scans were acquired to detect changes in glial activation (neuroinflammation) and glucose metabolism, respectively. The open-field test (OFT) was performed on days 6 and 27 to assess behavioral changes.

**Results:**

Twenty-five-gray-irradiated rats showed higher [^11^C]PK11195 uptake in most brain regions than controls on day 3 (striatum, hypothalamus, accumbens, septum *p* < 0.05), although some brain regions had lower uptake (cerebellum, parietal association/retrosplenial visual cortex, frontal association/motor cortex, somatosensory cortex, *p* < 0.05). On day 31, several brain regions in 25-Gy-irradiated rats still showed significantly higher [^11^C]PK11195 uptake than controls and 10-Gy-irradiated group (*p* < 0.05). Within-group analysis showed that [^11^C]PK11195 uptake in individual brain regions of 25-Gy treated rats remained stable or slightly increased between days 3 and 31. In contrast, a significant reduction (*p* < 0.05) in tracer uptake between days 3 and 31 was found in all brain areas of controls and 10-Gy-irradiated animals. Moreover, 10-Gy treatment led to a significantly higher [^18^F]FDG uptake on day 3 (*p* < 0.05). [^18^F]FDG uptake decreased between days 3 and 31 in all groups; no significant differences between groups were observed anymore on day 31, except for increased uptake in the hypothalamus in the 10-Gy group. The OFT did not show any significant differences between groups.

**Conclusions:**

Non-invasive PET imaging indicated that brain irradiation induces neuroinflammation and a metabolic flare, without causing acute or early-delayed behavioral changes.

**Electronic supplementary material:**

The online version of this article (10.1007/s11307-020-01483-y) contains supplementary material, which is available to authorized users.

## Introduction

Cranial irradiation is a frequently applied treatment modality for primary brain tumors and brain metastases, and as prophylactic treatment to prevent brain metastases. Despite its undisputed therapeutic importance, irradiation of normal brain tissue can lead to complications [1–4]. Depending on the time of onset, complications (intended as clinically apparent and tissue reaction) can be classified into (i) acute (days to weeks: sickness, nausea, vomiting, edema); (ii) early-delayed (1–4 months: neuroinflammation, transient demyelination, somnolence, cognitive deficits); and (iii) late-delayed (4–6 months to 1 year: neuroinflammation, vascular abnormalities, demyelination, radiation necrosis, irreversible cognitive decline) [3].

Neuroinflammation appears to be an important mechanistic link in the cascade leading towards delayed complications [5–7]. Neurons damaged by ionizing radiation release various chemokines, cytokines, and purine metabolites that activate microglia [8]. Activated microglia in turn secrete a panel of pro-inflammatory cytokines, which disrupt neurogenic signaling and neurogenesis, meanwhile stimulating infiltration of T lymphocytes and peripheral monocytes/macrophages [9] and inducing astrocyte activation, leading to gliosis and glial scar formation [10]. Selective inhibition of microglia-mediated neuroinflammation was shown to alleviate radiation-induced cognitive impairment [11].

Another radiation-induced adverse effect is decreased glucose metabolism. Radiotherapy was found to reduce the glucose metabolic rate in long-term survivors of childhood cancer [12, 13]. Indeed, radiation damage to normal cells causes electrophysiological and biochemical alterations, alterations in redox-sensitive processes, and direct mitochondrial damage resulting from the inflammatory response, which can all decrease cellular metabolism. Metabolic changes are likely to precede any anatomic changes like atrophy or radionecrosis [14].

In recent years, there has been a growing awareness that neuroimaging may be used to investigate early physiological effects after radiotherapy, thus helping to clarify the mechanisms underlying radiation induced-brain injury [15–19]. Positron emission tomography (PET) offers the opportunity to noninvasively measure physiological processes like neuroinflammation and glucose metabolism. Radiolabeled ligands targeting the 18-kDa translocator protein (TSPO) are tools to detect glial activation [20, 21]. TSPO expression is increased in activated microglia, activated astrocytes, infiltrating/perivascular macrophages, and T lymphocytes. TSPO overexpression is considered an *in vivo* biomarker for neuroinflammation and can be measured with 1-(2-chlorophenyl)-N-[^11^C]methyl-(1-methylpropyl)-3-isoquinoline carboxamide ([^11^C]PK11195). Brain glucose metabolism can be assessed with 2-deoxy-2-[^18^F]fluoro-d-glucose ([^18^F]FDG).

The aim of this study was to investigate the dose dependency of brain irradiation on acute and early-delayed glial cell activation (neuroinflammation) and changes in cerebral glucose metabolism using PET imaging. Since such molecular changes could culminate into behavioral abnormalities, our secondary aim was to assess whether our molecular imaging findings could be linked to behavioral changes assessed by the open-field test (OFT).

## Materials and Methods

### Experimental Animals

Male Wistar–Unilever rats (weight 300 ± 20 g; Harlan) were housed in groups of 2–4 in Makrolon cages with a layer of wood shavings in a room with constant temperature (21 ± 2 °C) and fixed 12-h light–dark regime. Standard laboratory chow (RMH-B, Hope Farms) and water were available *ad libitum*. After 7 days of acclimatization and 3 days of handling, the whole brain was irradiated with an X-RAD 320 apparatus (Precision X-Ray Inc.), using a homemade collimator (see Electronic Supplementary Materials).

### Study Design

**Fig. 1.**
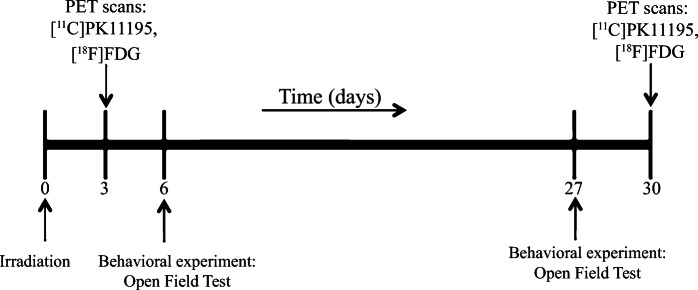
Study design.

Rats were randomly divided into 3 groups: (1) sham-irradiated controls (CTRL, *n* = 8); (2) irradiated with 10 Gy (10 Gy, *n* = 8); and (3) irradiated with 25 Gy (25 Gy, *n* = 8) of X-rays (Fig. 1). All animals underwent [^11^C]PK11195 and [^18^F]FDG PET scans on days 3 and 31. After the last PET scan, rats were sacrificed. Behavioral tests (OFT) were performed on days 6 and 27. The study was approved by the Animal Ethics Committee of the University of Groningen (DEC 6158A).

### PET Procedures

The [^11^C]PK11195 and [^18^F] FDG PET scans were performed on the same day for the two investigated time points (days 3 and 31 post irradiation). Rats were anesthetized with isoflurane (5 % induction, 1.5–2 % maintenance). In the morning, [^11^C]PK11195 was injected *via* the penile vein (32 ± 20 MBq), and rats were allowed to wake up in their cage. After 40 min, rats were anesthetized again and positioned in a prone position in the PET camera (microPET Focus 220; Siemens Medical Solutions) with the brain in the field of view. Eye-salve was applied, body temperature was maintained with heating pads, and oxygen saturation was monitored. A 30-min emission scan was started 45 min after tracer injection, followed by a 5-min transmission scan with a Co-57 point source for attenuation and scatter correction. At least 2 h after completion of the [^11^C]PK11195 scan, [^18^F]FDG was intraperitoneally injected in awake rats (20 ± 8 MBq) to allow tracer distribution to the brain while the animal is active, according to the standard procedure in our department [22, 23, 24, 25]. A [^18^F]FDG PET scan was acquired as described above for [^11^C]PK11195 PET. The remaining radioactivity derived from [^11^C]PK11195 was negligible (> 12 half-lives; < 0.02 %) at the time of the [^18^F]FDG PET scan.

Scans were iteratively reconstructed (OSEM2D, 4 iterations, and 16 subsets) into a single frame of 30 min and corrected for attenuation, scatter, random coincidences, and radioactive decay. The images had a matrix of 128 × 128 × 95 mm, 0.475-mm pixel width, and 0.796-mm slice thickness and were analyzed using PMOD-3.8 software (PMOD Technologies Ltd). The scans were automatically registered to tracer-specific PET templates [26]. Volumes of interest (VOIs) were constructed for several previously defined brain regions [26]. The radioactivity concentration was calculated for each VOI and converted into percentage of injected dose/gram (%ID/g), which are defined as follows: (radioactivity concentration in tissue [Bq/cm^3^] × 100%)/(injected dose [Bq]). All data presented was obtained from image analysis; no *ex vivo* biodistribution was performed.

### Behavioral Study

The open-field test (OFT) was performed during the light phase. The rat was placed in the center of an ellipsoid arena (126 × 88 cm) and allowed to explore for 5 min. Animal behavior was recorded on video. The videos were analyzed with EthoVision XT9 software (Noldus Information Technology, Wageningen) and total distance moved was analyzed.

### Statistical Analysis

All data is presented as mean ± standard deviation. Statistical analysis was performed using IBM SPSS Statistics-23. Between-group comparisons were used to detect the effect of whole-brain irradiation on body weight, [^11^C]PK11195 uptake, [^18^F]FDG uptake, and behavior for individual time points. Within-group comparisons were used to assess time-dependent changes in the above-mentioned parameters. Between-group and within-group differences were analyzed using the generalized estimated equation model using the pairwise comparison option. A *p* value ≤ 0.05 was considered statistically significant.

## Results

### Body weight

**Fig. 2.**
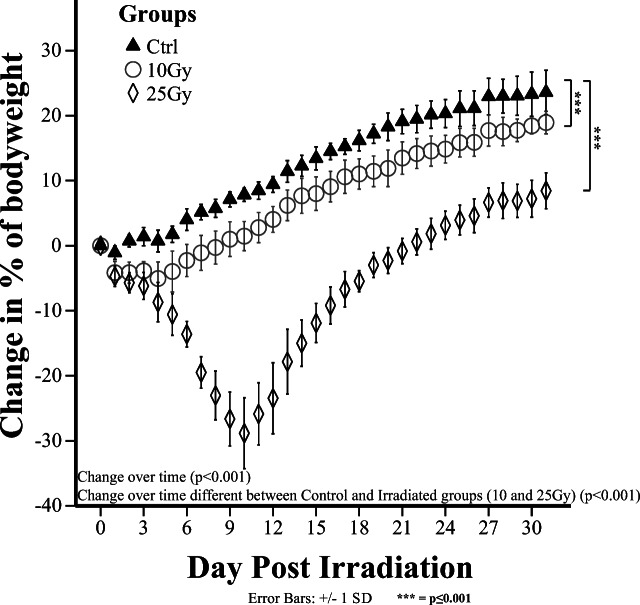
Effects of whole-brain irradiation on body weight. Both 10-Gy and 25-Gy irradiation caused a significant (*p* < 0.001) reduction in body weight (*n* = 8 rats per group).

Body weight was measured daily to assess the effect of brain irradiation (Fig. 2). Statistically significant (*p* < 0.001) effects were observed for the factors “time” and “group.” Irradiation with either 10 Gy or 25 Gy of X-rays caused a significant reduction in body weight as compared with controls (*p* < 0.001). Both control and 10 Gy rats showed a gradual increase in body weight throughout the experiment. In contrast, rats receiving 25 Gy showed a continuous weight loss during the first 10 days (− 22.8 % compared with day 0) and a subsequent weight gain, without reaching the body weight of the control and 10 Gy group.

### [^11^C]PK11195 PET

A total of 8 rats per group were used, but not all were included in the [^11^C]PK11195 analysis because of tracer injection failure. As such, one control rat and two rats irradiated with 10 Gy were excluded for the analysis of day 3, and one rat irradiated with 25 Gy for the analysis of day 31.

**Fig. 3.**
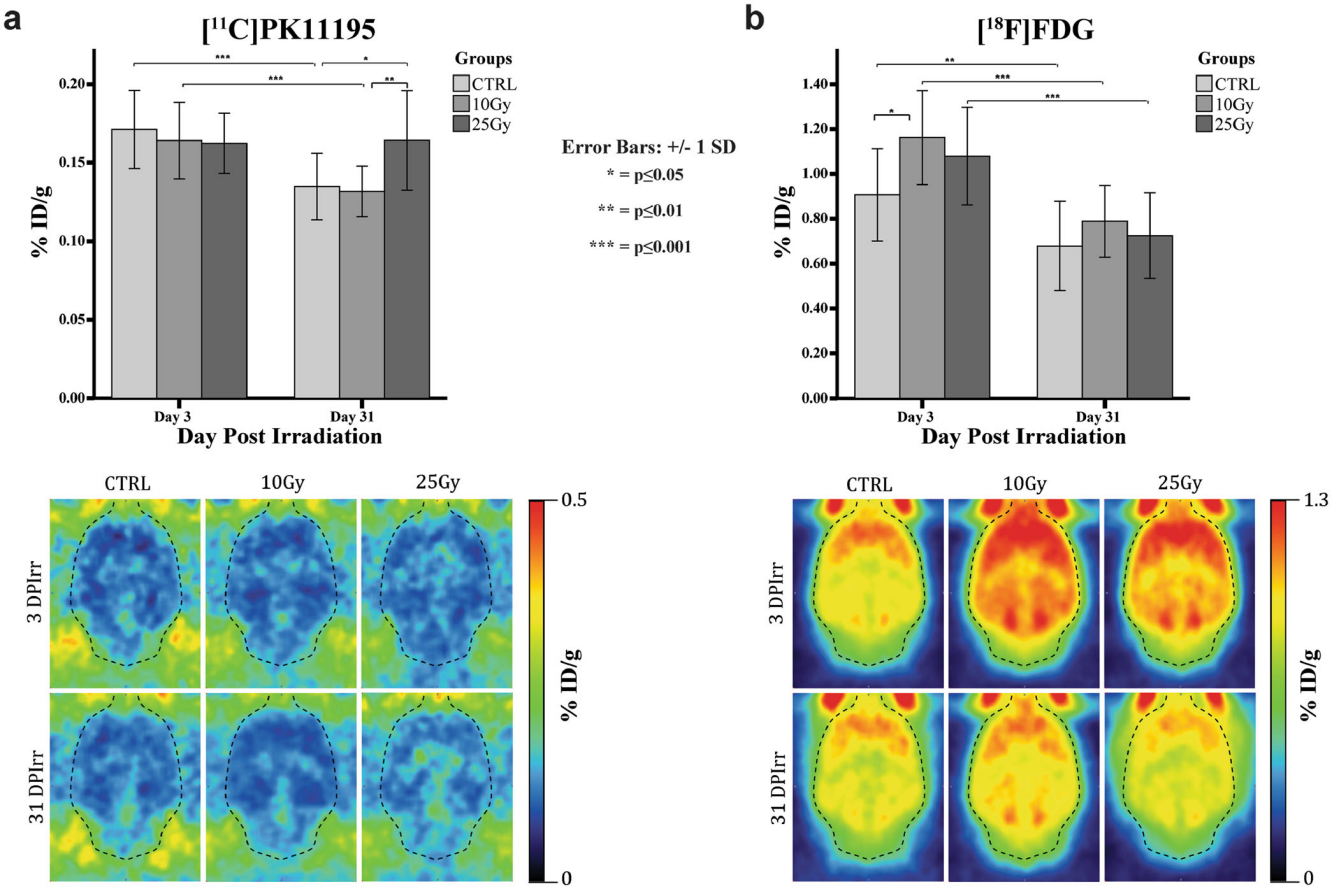
**a** Whole-brain [^11^C]PK11195 uptake, expressed as % injected dose/gram, on days 3 and 31 after sham (*n* = 7 and *n* = 8), 10-Gy (*n* = 6 and *n* = 8), or 25-Gy (*n* = 8 and *n* = 7) whole-brain irradiation. **b** Whole-brain [^18^F]FDG uptake, expressed as % injected dose/gram (mean ± SD), on days 3 and 31 after sham (*n* = 7 and *n* = 8), 10-Gy (*n* = 8 and *n* = 8), or 25-Gy (*n* = 8 and *n* = 8) whole-brain irradiation.

As shown in Fig. 3a, whole-brain [^11^C]PK11195 uptake on day 3 was comparable in all groups (10 Gy, 0.16 ± 0.02 %ID/g; 25 Gy, 0.16 ± 0.02 %ID/g; CTRL, 0.17 ± 0.02 %ID/g). Whole-brain [^11^C]PK11195 uptake in the 25 Gy group did not significantly change between days 3 and 31 (+ 1.5 %, *p* = 0.84), whereas whole-brain uptake in controls (− 21.2 %; *p* ≤ 0.001) and the 10 Gy group (− 19.7 %; *p* ≤ 0.001) significantly decreased in this period. Consequently, a significant difference in [^11^C]PK11195 uptake was observed on day 31 between the 25 Gy group (0.16 ± 0.03 %ID/g) and the 10 Gy group (0.13 ± 0.016 %ID/g; *p* = 0.008) and between the 25 Gy group and controls (0.13 ± 0.021 %ID/g; *p* = 0.025).

Individual brain regions showed in general similar trends in [^11^C]PK11195 uptake as whole brain, with some exceptions (Table 1). On day 3, for example, in contrast to the whole-brain value, a significant reduction in [^11^C]PK11195 uptake compared with controls was observed in frontal association and motor cortex in the 10 Gy group. Meanwhile, in the 25 Gy group, significant reductions were found in the cerebellum, frontal association, motor, somatosensory, parietal association, and retrosplenial and visual cortex compared with controls. In contrast, a significant increase [^11^C]PK11195 uptake compared with controls was observed in the septum in the 10 Gy group and in the striatum, hypothalamus, accumbens, and septum in the 25 Gy group. Within-group analysis revealed a significant reduction in [^11^C]PK11195 uptake in all individual brain regions in the control and 10 Gy group between days 3 and 31, except for septum in controls. In contrast, the 25 Gy group only showed a significant reduction in tracer uptake in the insula and auditory and temporal association cortex. Consequently, no significant differences in any brain region between the control and 10 Gy group were observed on day 31. In contrast, on day 31, the 25 Gy group showed a significantly higher [^11^C]PK11195 uptake in all individual brain regions than controls, with the exception of the cerebellum, entorhinal olfactory, frontal association, and motor cortex. On day 31, [^11^C]PK11195 uptake in all brain regions in the 25 Gy group was also significantly higher than in the 10 Gy group, except for the cerebellum, insula, amygdala, and entorhinal olfactory.

**Table 1.** [^11^C]PK11195 uptake, expressed as % injected dose/gram (mean ± SD), in different rat brain regions, at different irradiation dose, at 3 and 31 days after whole-brain (sham-)irradiation

Time point ➔	Day 3			Day 31		
Group➔	CTRL (*n* = 7)	10 Gy (*n* = 6)	25 Gy (*n* = 8)	CTRL (*n* = 8)	10 Gy (*n* = 8)	25 Gy (*n* = 7)
Whole brain	0.17 ± 0.02	0.16 ± 0.02	0.16 ± 0.02	0.13 ± 0.02^#^	0.13 ± 0.02^#^	0.16 ± 0.03*^,^^
Cerebellum	0.22 ± 0.03	0.20 ± 0.03	0.18 ± 0.03*	0.18 ± 0.03^#^	0.17 ± 0.03^#^	0.18 ± 0.03
Frontal association and motor cortex	0.24 ± 0.05	0.19 ± 0.03 *	0.19 ± 0.03*	0.18 ± 0.04^#^	0.16 ± 0.03^#^	0.21 ± 0.04^^^
Somatosensory cortex	0.19 ± 0.03	0.17 ± 0.03	0.16 ± 0.02*	0.14 ± 0.03^#^	0.14 ± 0.02^#^	0.17 ± 0.03*^,^^
Parietal asso. + retrosplenial + visual cortex	0.22 ± 0.04	0.20 ± 0.03	0.19 ± 0.03*	0.19 ± 0.04^#^	0.17 ± 0.03^#^	0.20 ± 0.03^^^
Striatum	0.11 ± 0.02	0.13 ± 0.02	0.14 ± 0.01*	0.09 ± 0.02^#^	0.10 ± 0.01^#^	0.14 ± 0.03*^,^^
Thalamus	0.12 ± 0.02	0.13 ± 0.02	0.14 ± 0.02	0.11 ± 0.02^#^	0.11 ± 0.01^#^	0.14 ± 0.03*^,^^
Hypothalamus	0.13 ± 0.02	0.14 ± 0.02	0.15 ± 0.02*	0.11 ± 0.02^#^	0.10 ± 0.01^#^	0.15 ± 0.03*^,^^
Hippocampus	0.13 ± 0.02	0.13 ± 0.02	0.14 ± 0.01	0.11 ± 0.02^#^	0.11 ± 0.01^#^	0.14 ± 0.02*^,^^
Brainstem	0.16 ± 0.02	0.17 ± 0.02	0.16 ± 0.02	0.13 ± 0.02^#^	0.13 ± 0.01^#^	0.18 ± 0.05*^,^^
Midbrain	0.13 ± 0.02	0.14 ± 0.02	0.14 ± 0.02	0.11 ± 0.02^#^	0.11 ± 0.01^#^	0.15 ± 0.04*^,^^
Cingulate	0.15 ± 0.03	0.14 ± 0.03	0.16 ± 0.03	0.13 ± 0.02^#^	0.11 ± 0.02^#^	0.16 ± 0.02*^,^^
Accumbens	0.12 ± 0.02	0.13 ± 0.02	0.14 ± 0.02*	0.09 ± 0.01^#^	0.10 ± 0.01^#^	0.14 ± 0.03*^,^^
Prefrontal + orbital cortex	0.16 ± 0.02	0.16 ± 0.03	0.16 ± 0.02	0.12 ± 0.02#	0.12 ± 0.02^#^	0.15 ± 0.02*^,^^
Septum	0.12 ± 0.02	0.15 ± 0.01*	0.17 ± 0.02*^,^^	0.11 ± 0.02^#^	0.11 ± 0.01^#^	0.16 ± 0.02*^,^^
Insula	0.16 ± 0.03	0.14 ± 0.03	0.16 ± 0.02	0.12 ± 0.02^#^	0.12 ± 0.02^#^	0.14 ± 0.03*^,#^
Auditory + temporal asso. cortex	0.17 ± 0.02	0.16 ± 0.03	0.17 ± 0.02	0.12 ± 0.02^#^	0.13 ± 0.01^#^	0.15 ± 0.03*^,^,#^
Amygdala	0.16 ± 0.02	0.16 ± 0.03	0.16 ± 0.02	0.12 ± 0.02^#^	0.12 ± 0.01^#^	0.16 ± 0.05*
Entorhinal olfactory	0.17 ± 0.03	0.17 ± 0.02	0.17 ± 0.02	0.13 ± 0.02^#^	0.16 ± 0.01^#^	0.16 ± 0.05

Statistically significant difference between groups at the same time point: *p ≤ 0.05 25 Gy or 10 Gy *vs* CTRL; ^^^*p* ≤ 0.05 25 Gy *vs* 10 Gy

Statistically significant reduction between time points: ^#^*p* ≤ 0.05 day 3 *vs* day 31

### [^18^F]FDG PET

A total of 8 rats per group were used, but not all were included in the [^18^F]FDG analysis because of tracer injection failure. As such, one control rat was excluded for the analysis of day 3.

As shown in Fig. 3b, whole-brain [^18^F]FDG uptake on day 3 was significantly higher in rats irradiated with 10 Gy (1.16 ± 0.21 %ID/g, *p* = 0.011) than in controls (0.91 ± 0.21 %ID/g). Tracer uptake in the 25 Gy group (1.08 ± 0.22 %ID/g) on day 3 was also higher than in controls, but this difference was not statistically significant (*p* = 0.091). In all groups, whole-brain tracer uptake significantly decreased between day 3 and 31 (intragroup: 10 Gy, − 32.1 %, *p* ≤ 0.001; 25 Gy, − 32.8 %, *p* ≤ 0.001; CTRL, − 25.2 %, *p* = 0.011). In general, all individual brain regions showed similar [^18^F]FDG uptake changes as shown for the whole brain (Table 2). In particular, all brain regions in the 10 Gy group showed significantly higher [^18^F] FDG uptake than controls on day 3, whereas none of the brain regions in the 25 Gy group showed any significant difference in tracer uptake as compared with controls. On day 31, none of the individual brain regions showed any significant difference in [^18^F]FDG uptake between groups, except for the hypothalamus in the 10 Gy group, which showed significantly higher [^18^F]FDG uptake than controls.

**Table 2:** [^18^F]FDG uptake, expressed as % injected dose/g (mean ± SD), in different rat brain regions, at different irradiation dose, at 3 and 31 days after whole-brain (sham-)irradiation

Time point ➔	Day 3			Day 31		
Group ➔	CTRL (*n* = 7)	10 Gy (*n* = 8)	25 Gy (*n* = 8)	CTRL (*n* = 8)	10 Gy (*n* = 8)	25 Gy (*n* = 8)
Whole brain	0.91 ± 0.21	1.16 ± 0.21*	1.08 ± 0.22	0.68 ± 0.20^#^	0.79 ± 0.16^#^	0.73 ± 0.19^#^
Cerebellum	0.76 ± 0.18	1.00 ± 0.18*	0.92 ± 0.19	0.58 ± 0.16^#^	0.71 ± 0.13^#^	0.61 ± 0.15^#^
Frontal association and motor cortex	0.79 ± 0.19	0.98 ± 0.18*	0.91 ± 0.17	0.59 ± 0.18^#^	0.66 ± 0.13^#^	0.60 ± 0.16^#^
Somatosensory cortex	0.88 ± 0.20	1.11 ± 0.19*	1.05 ± 0.21	0.66 ± 0.18^#^	0.73 ± 0.15^#^	0.70 ± 0.20^#^
Parietal asso. + retrosplenial + visual cortex	0.82 ± 0.19	1.04 ± 0.18*	0.98 ± 0.19	0.58 ± 0. 81^#^	0.69 ± 0.13^#^	0.61 ± 0.15^#^
Striatum	1.10 ± 0.22	1.39 ± 0.26*	1.27 ± 0.26	0.81 ± 0.26^#^	0.94 ± 0.20^#^	0.88 ± 0.25^#^
Thalamus	1.03 ± 0.23	1.35 ± 0.26*	1.24 ± 0.27	0.76 ± 0.25^#^	0.92 ± 0.21^#^	0.84 ± 0.24^#^
Hypothalamus	0.80 ± 0.19	1.05 ± 0.22*	0.98 ± 0.21	0.58 ± 0.20^#^	0.75 ± 0.15^#,^*	0.64 ± 0.16^#^
Hippocampus	1.01 ± 0.23	1.27 ± 0.24*	1.19 ± 0.25	0.75 ± 0.24^#^	0.87 ± 0.19^#^	0.80 ± 0.21^#^
Brainstem	0.82 ± 0.22	1.08 ± 0.22*	0.99 ± 0.19	0.64 ± 0.16^#^	0.77 ± 0.15^#^	0.66 ± 0.16^#^
Midbrain	0.96 ± 0.24	1.28 ± 0.25*	1.18 ± 0.24	0.72 ± 0.24^#^	0.88 ± 0.19^#^	0.80 ± 0.20^#^
Cingulate	1.10 ± 0.26	1.35 ± 0.24*	1.29 ± 0.24	0.81 ± 0.26^#^	0.91 ± 0.19^#^	0.82 ± 0.22^#^
Accumbens	1.08 ± 0.24	1.37 ± 0.25*	1.26 ± 0.28	0.79 ± 0.26^#^	0.92 ± 0.19^#^	0.86 ± 0.23^#^
Prefrontal + orbital cortex	1.15 ± 0.26	1.44 ± 0.24*	1.34 ± 0.27	0.87 ± 0.27^#^	0.95 ± 0.20^#^	0.93 ± 0.24^#^
Septum	0.99 ± 0.22	1.26 ± 0.24*	1.17 ± 0.25	0.70 ± 0.24^#^	0.85 ± 0.18^#^	0.78 ± 0.23^#^
Insula	0.94 ± 0.21	1.18 ± 0.19*	1.12 ± 0.24	0.74 ± 0.20^#^	0.77 ± 0.16^#^	0.84 ± 0.27^#^
Auditory + temporal asso. cortex	0.87 ± 0.20	1.13 ± 0.21*	1.03 ± 0.21	0.67 ± 0.17^#^	0.76 ± 0.17^#^	0.74 ± 0.23^#^
Amygdala	0.87 ± 0.19	1.12 ± 0.21*	1.04 ± 0.22	0.66 ± 0.20^#^	0.74 ± 0.15^#^	0.70 ± 0.19^#^
Entorhinal olfactory	0.90 ± 0.20	1.15 ± 0.20*	1.08 ± 0.22	0.67 ± 0.20^#^	0.77 ± 0.16^#^	0.72 ± 0.18^#^

Statistically significant difference between groups at the same time point: *p ≤ 0.05 25 Gy or 10 Gy *vs* CTRL

Statistically significant reduction between time points: ^#^*p* ≤ 0.05 day 3 *vs* day 31

### Behavioral Assessment

**Fig. 4.**
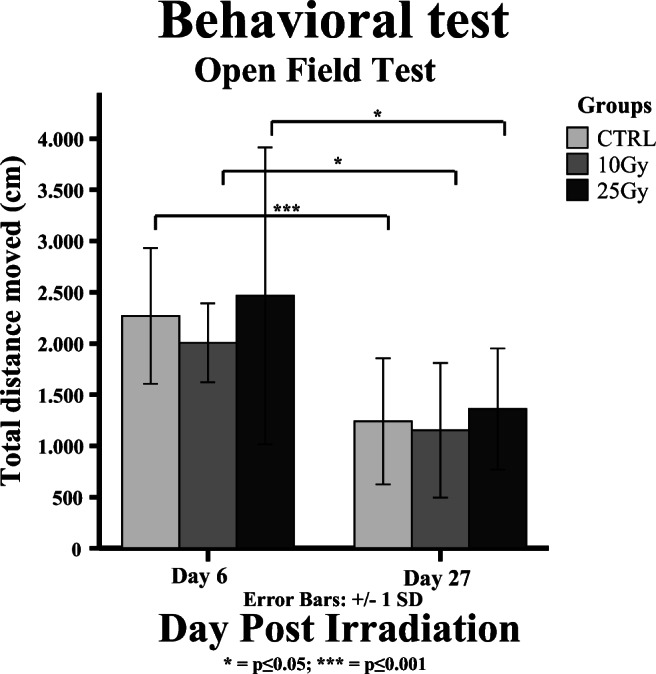
Open-field test, showing no significant differences between the experimental groups, but a significant difference between the two time points (control group, *p* < 0.001; 10 Gy group, *p* = 0.021; 25 Gy group *p* = 0.045; *n* = 8).

In the open-field test on day 6, no significant differences in total distance moved were observed between groups (Fig. 4). Animals from all groups moved significantly less on day 27 than on day 6 (control, − 45 %, *p* < 0.001; 10 Gy, − 43 %, *p* = 0.021; 25 Gy, − 45 %, *p* = 0.045), but no significant differences between experimental groups were observed.

## Discussion

The primary aim of this study was to investigate the acute and early-delayed, dose-dependent effects of brain irradiation on glial cell activation and cerebral glucose metabolism. We observed that whole-brain irradiation in healthy rats caused a dose-dependent reduction in body weight; a transient metabolic flair in the brain, especially in the 10Gy group; and a delayed neuroinflammatory response, in particular in the high-dose group.

The radiolabeled TSPO ligand [^11^C]PK11195 was selected as *in vivo* brain inflammation marker [22, 27], because TSPO expression is increased in activated microglia/astrocytes, infiltrating/perivascular macrophages/lymphocytes, and infiltrating neutrophils in response to neuroinflammatory stimuli. Radiotherapy can induce damage to the brain, and stress signals from damaged cells can trigger the activation of glial cells, which is accompanied by an increase in TSPO expression. Moreover, radiotherapy can induce the production of reactive oxygen species (ROS). TSPO is known to form polymers in the presence of ROS and these polymers bind [^11^C]PK11195 with higher affinity than the monomer [28]. For these reasons, we expected cranial irradiation to increase [^11^C]PK11195 binding in the brain. Although whole-brain [^11^C]PK11195 uptake in the acute phase was not significantly different between groups, to our surprise, whole-brain irradiation caused an acute, transient decrease in [^11^C]PK11195 accumulation in the cerebellum and several cortical regions in the 25 Gy group. It is known that, under normal physiological conditions, low levels of TSPO expression can be detected in glial cells, but also in other cells in the brain, such as cerebrovascular/endothelial cells, and smooth muscle cells [29, 30]. The TSPO-expressing cells in the brain can be damaged by high doses of radiation and go into apoptosis. A possible explanation for the acute reduction in the [^11^C]PK11195 signal in these brain regions early after irradiation could therefore be a temporary reduction in the number of glial and/or endothelial cells due to radiation-induced cell death [31–34]. This result is in agreement with a flow cytometry study in mice that demonstrated that the number of CD11b-expressing microglia was significantly reduced 7 and 14 days after cranial radiation (10Gy), but had recovered to control levels on day 28 [33]. Besides brain regions with reduced [^11^C]PK11195 uptake, the 25 Gy group also has regions in the brain with increased tracer uptake on day 3. The regions with increased tracer uptake are more centrally located than the regions with reduced tracer uptake. So, an alternative explanation for our [^11^C]PK11195 PET findings on day 3 could be glial cells migrating from cortical brain regions to the midbrain in response to 25-Gy irradiation.

On day 31, our study showed that [^11^C]PK11195 uptake was significantly higher in almost all brain regions in the 25-Gy-irradiated animals than in controls, with the exception of the cerebellum and some cortical areas. Interestingly, these exceptions were the brain regions that had shown reduced tracer uptake on day 3. These data suggest a delayed activation of glial cells in most brain regions and a normalization of the concentration of glial cells in regions that were depleted of TSPO-expressing cells on day 3 after the acute phase.

In contrast to the 25-Gy group, rats that were irradiated with 10 Gy had [^11^C]PK11195 uptake similar to that in controls in almost all regions, both on days 3 and 31. Apparently, the lower irradiation dose is not sufficient to induce any detectable glial activation. Dose-dependent differences in the severity of the radiation-induced tissue damage also seem to be reflected in the effect of brain irradiation on body weight, as the high 25-Gy dose caused significant weight loss (Fig. 1), whereas the lower 10-Gy dose had a much smaller effect on body weight. Similar effects of cranial irradiation on rodent body weight have previously been reported [35, 36].

Noteworthy, the decline in [^11^C]PK11195 uptake between days 3 and 31 in control and 10 Gy treated could be pointing to a combination of repeated animal handling and stress induced to them in a short time due to the experimental procedures, *i.e.*, pre-whole-brain (sham-)irradiation anesthesia by intraperitoneal injection, (sham-)irradiation procedures (rats hanging by upper incisors for about 18 min), the slow recovery from anesthesia, and transport to the PET center, as well the xylazine/ketamine administration for the irradiation procedure. Indeed, a single administration of ketamine increased the protein levels of IL-6, IL-1β, and TNF-α, in mice brain 6 h post intraperitoneal injection [37].

Taken together, all the procedures before the first PET scan could stress the animals resulting in a microglia imprinting that leads to some inflammatory response in the brain that gradually disappeared, or was not detectable anymore on day 31, in healthy and 10-Gy irradiated animals. This is also in line with the higher [^18^F]FDG value found on day 3 *vs* 31, where the inflammatory response could increase [^18^F]FDG uptake on day 3 [38]. That this decline was not observed in the rats irradiated with 25Gy might be related to an inflammatory response to the irradiation, resulting in increased [^11^C]PK11195 uptake compensating the decline observed in the control and 10-Gy-treated group.

Whole-brain irradiation increased brain glucose metabolism on day 3. This could be ascribed to an early adaptive response of the brain to meet the energy demand for repair of radiation-induced tissue damage [39] or induction of GLUT1 transporter expression [40]. Remarkably, the effect of cranial irradiation on glucose metabolism was more pronounced in the 10-Gy group than in the 25-Gy group. This difference in metabolic response might be explained by the extent of damage induced by the treatment [41]. Radiation induces a peak in apoptosis 6 h after irradiation, and apoptosis continues for another 24–48 h, while radiation also can increase cell proliferation between 1 and 7 days after irradiation [42, 43]. It seems plausible that the impact of apoptosis is more important in the 25-Gy group than in the 10-Gy group, resulting in a smaller net energy demand for repair and thus a smaller increase in glucose metabolism in the high-dose group. Between days 3 and 31, cerebral [^18^F]FDG uptake in all groups decreased. This reduction in cerebral glucose metabolism may be due to a general reduction in activity of the rats upon aging. This is in line with our findings of reduced mobility in the OFT.

The PET data suggest that whole-brain irradiation causes a transient “flare” response of glucose metabolism, in combination with delayed activation of glial cells in the brain. These physiological effects, however, did not seem to be associated with behavioral changes as assessed by the OFT. The OFT tests did not show any significant radiation-induced behavioral differences between control and irradiated animals. So, either the functional changes observed by PET are too subtle to induce behavioral changes, or the statistical power of the experiment was insufficient to reveal the subtle behavioral changes. In general, inter-individual variability in behavioral studies is larger than that in PET studies. Consequently, a larger group size would be required for behavioral tests. Other studies, however, have shown late behavioral changes after cranial irradiation [44, 45]. It is possible that other types of behavioral changes (*e.g.*, impairment cognition) were induced than could be detected in the OFT. Therefore, future research should aim to correlate PET imaging with late behavioral changes, in particular with more appropriate behavioral tests, like memory tests such as Y-maze, Morrison maze, or novel object/spatial recognition tests. The OFT in this study did show a reduction in locomotion between days 6 and 27 in all groups, but no differences between groups. The reduction in exploratory behavior between both time points could be ascribed to recognition of the arena in the repeated test. Moreover, animals tend to move less when they get older. The latter explanation is in line with the general reduction in glucose metabolism in all groups observed by [^18^F]FDG PET, suggesting a general reduction in activity.

A limitation of our study is that imaging findings were not confirmed by *ex vivo* histology or immunohistochemistry, due to the longitudinal study design. Another limitation is the lack of cell-type (*e.g.*, microglia, macrophage, astrocyte) and phenotype specificity (M1, M2) of [^11^C]PK11195 PET. Attempts to develop more specific tracers are in progress. Finally, the sample size in this study may have been too small to reveal statistically significant behavioral changes, and the open-field behavioral test may not have been suitable to demonstrate radiation-induced cognitive impairment.

## Conclusion

Taken together, this study has demonstrated dose-dependent acute and early-delayed changes in cerebral physiology after radiotherapy. PET proved an adequate method to monitor these changes in a longitudinal manner, as non-invasive PET imaging revealed that whole-brain irradiation of healthy rats induces a transient metabolic flare and delayed neuroinflammation (glial activation), without causing acute or early-delayed behavioral changes. Additional studies, using different (fractionated) dosing schemes, are required to evaluate the impact of the observed physiological changes on the development of late-delayed clinical complications and the mechanisms that are involved in this process.

## Electronic Supplementary Material

ESM 1 (DOCX 574 kb).
