# Supplementary material for: The Acute and Early Effects of Whole-Brain Irradiation on Glial Activation, Brain Metabolism, and Behavior: a Positron Emission Tomography Study
Source: Mol Imaging Biol. 2020 Feb 12;22(4):1012–20. doi: 10.1007/s11307-020-01483-y (PMC7343765; doi:10.1007/s11307-020-01483-y)
Supplement: Supplementary file 1 — (DOCX 574 kb). [file 11307_2020_1483_MOESM1_ESM.docx]

**Electronic Supplementary Material**

**The Acute and Early Effects of Whole Brain Irradiation on Glial Activation, Brain Metabolism and Behavior: a Positron Emission Tomography Study**

**Journal: Molecular Imaging and Biology**

Andrea Parente,^1^ Erik F.J. de Vries, ^1^ Aren van Waarde,^1^ Magdalini Ioannou,^1^ Peter van Luijk,^2,3^ Johannes A. Langendijk,^3^ Rudi A.J.O. Dierckx,^1^ Janine Doorduin ^1^

^1^ Department of Nuclear Medicine and Molecular Imaging, University of Groningen, University Medical Center Groningen, Hanzeplein 1, 9713 GZ Groningen, The Netherlands

^2^ Department of Cell Biology, University of Groningen, University Medical Center Groningen, Groningen, The Netherlands.

^3^ Department of Radiation Oncology, University of Groningen, University Medical Center Groningen, Groningen, The Netherlands.

**Correspondence to**:

Prof. Erik F.J. de Vries; *e-mail:* e.f.j.de.vries@umcg.nl.

Department of Nuclear Medicine and Molecular Imaging, University of Groningen, University Medical Center Groningen, P.O. Box 30.001, 9700 RB Groningen, The Netherlands.

Tel.: +31-50-3613599 / Fax: +31-50-3611687

**Supplementary Material**

### *Whole Brain Irradiation procedure*

Whole brain irradiation was performed with a X-RAD 320 apparatus (Precision X-Ray Inc.), operated at 200-kV and 20-mA tube current. A protocol for absolute dosimetry was designed, and relative-dose measurements were performed. Dose–volume histograms were determined by the three-dimensional (3D) topography data and the 3D dose distributions. Rats were anesthetized by intraperitoneal injection of a mixture of Rompun (xylazine; Bayer AG, Leverkusen, Germany; 0.48 mg/100 g bodyweight) and Ketalar (S-ketamine, Pfizer BV; Capelle aan de IJssel, The Netherlands; 3.25 mg/100 g bodyweight). After at least 10-15min, the rats were positioned in a holder, more precisely, they were placed in a groove of the rod, to standardize positioning and guarantee an anatomically correct irradiation area (Fig.1C) [1]. The dorsal part of the animals rested against a dedicated collimator with an opening on the top of the brain (Fig.1B). The irradiation holder was placed on a platform (pin and hole construction) attached to the beam exit window (bayonet-ring construction) of the X-ray tube. The platform was designed in such a way that the central beam axis coincided with the z-axis of the brain to minimize the divergence of the beam. An additional 3-mm lead shielding was placed in front of caudal extremities of the rat, although these body parts were outside the X-ray beam and collimator. Experimental rats received a radiation dose of either 10 or 25Gy on their brain (1.559Gy/min, respectively 385 and 962 seconds of exposition). Control rats were similarly positioned in the holder, but the X-ray apparatus remained switched off.

**Figure 1** **Supplementary Material:** A) MRI-based outline of average rat brains and tear glands; B) The brain collimator, made of 3-mm lead (grey) embedded in a brass plate (yellow-brown); C) Holder with the collimator for highly reproducible irradiation of the rat brain.

### *Collimator design*

3D anatomical MRI images were acquired from 6 Wistar rats (280-320g) and imported in Osiris software in DICOM format. On the 3D images, regions-of-interest (ROIs) delineating the brain, the eyeballs and the tear glands, were drawn for different Z-coordinates (ventral to dorsal). A plot of the ROIs at a Z-level of about 5mm from the top of the rat skull (start point for the Z-coordinate of the multilayer 3D images) was made. Then, a new ROI of the brain was delineated that excluded the eye balls/tear glands at this Z-level with a margin of 2 mm. Next, the required portal outline of the collimator was calculated using a program written in Matlab, taking into account the divergence of the X-ray beam. This procedure was repeated for other Z values and resulted in an outline of the collimator opening for conformal radiation beams that irradiated the brain with minimal doses on normal tissues and tear glands (Fig.1). The calculated portal opening was cut into a 3-mm thick lead sheet. To ensure accurate fixation, the lead sheet was embedded in a brass plate (Fig. 1B). The collimator was mounted in a plastic holder containing a rod with a groove, in which the teeth of the rats could be placed (Fig. 1C). This allowed highly reproducible positioning of rats, ensuring administration of the correct dose to the appropriate brain volume throughout the experiment.

1. Cotteleer F, Faber H, Konings AWT, et al (2003) Three-dimensional dose distribution for partial irradiation of rat parotid glands with 200kV X-rays. Int J Radiat Biol 79:689–700
